# A CRISPR/Cas9‐based method for targeted DNA methylation enables cancer initiation in B lymphocytes

**DOI:** 10.1002/ggn2.10040

**Published:** 2021-03-11

**Authors:** Shota Katayama, Koichi Shiraishi, Naoki Gorai, Masao Andou

**Affiliations:** ^1^ IMRA Japan Co., Ltd. Sapporo Japan; ^2^ AISIN AW Co., Ltd. Anjou Japan

**Keywords:** cancer initiation, cancer risk evaluation, CRISPR/Cas9, DNA methylation, gene expression, MMEJ

## Abstract

Targeted DNA methylation is important for understanding transcriptional modulation and epigenetic diseases. Although CRISPR‐Cas9 has potential for this purpose, it has not yet been successfully used to efficiently introduce DNA methylation and induce epigenetic diseases. We herein developed a new system that enables the replacement of an unmethylated promoter with a methylated promoter through microhomology‐mediated end joining‐based knock‐in. We successfully introduced an approximately 100% DNA methylation ratio at the cancer‐associated gene SP3 in HEK293 cells. Moreover, engineered *SP3* promoter hypermethylation led to transcriptional suppression in human B lymphocytes and induced B‐cell lymphoma. Our system provides a promising framework for targeted DNA methylation and cancer initiation through epimutations.

## Graphical abstract

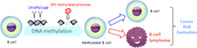

## 1 INTRODUCTION

Methods for targeted DNA methylation are needed to obtain a more detailed understanding of transcriptional modulation and epigenetic diseases. Small molecules, which induce DNA methylation, alter the epigenetic state globally, but cannot target specific loci. The clustered, regularly interspaced, short palindromic repeat (CRISPR)/CRISPR‐associated protein (Cas9) system has been shown to target specific genomic loci and induces site‐directed DNA breaks when combined with single‐guide RNA (sgRNA) containing the complementary 20 nucleotides for the target sequence.^1^, ^2^, ^3^, ^4^, ^5^, ^6^, ^7^, ^8^ Recent studies demonstrated that the fusion of effector domains or proteins to catalytically dead Cas9 (dCas9) extended applications to targeted epigenome editing, such as de novo DNA methylation through dCas9‐methyltrasferase fusion proteins.^9^, ^10^, ^11^, ^12^, ^13^, ^14^, ^15^, ^16^, ^17^, ^18^, ^19^ However, the introduction of an approximately 100% DNA methylation ratio at targeted loci and the induction of epigenetic diseases have not yet been achieved. Therefore, a novel system is needed.

We focused on the microhomology‐mediated end joining (MMEJ)‐dependent integration of donor DNA using CRISPR‐Cas9.^20^, ^21^ MMEJ requires a short homologous sequence (5‐25 bp) for DNA double‐strand break repair, resulting in precise integration into the targeted genomic loci.^22^, ^23^, ^24^, ^25^, ^26^, ^27^ MMEJ‐mediated precise integration enables the development of a DNA methylation system by which an activated gene is silenced through the replacement of an unmethylated promoter with a methylated promoter. This system may be used to induce epigenetic diseases.

More than 33 years ago, epimutation—gene silencing associated with epigenetic alterations in DNA methylation—was proposed to be necessary for tumorigenesis.^28^ Promoter CpG island‐associated genes were previously shown to be hypermethylated and silenced in many types of cancers.^29^ The majority of cancer types harbor hundreds of abnormally hypermethylated promoter CpG islands,^30^ indicating that epimutations are common in tumors. A previous study reported that epimutations in *p16* ^*INK4a*^, a tumor suppressor gene, in vivo induced lymphoma and sarcoma in mice.^31^ Furthermore, the premature termination of reprogramming (induced by *Oct‐3/4*, *Sox‐2*, *Klf‐4*, and *c‐Myc*) in vivo caused kidney tumors through epimutations in mice.^32^ However, it currently remains unclear whether epimutations induce human cancer cells in vitro.

Lymphoma and sarcoma are caused by epimutations in mice. Specificity protein 3 (SP3), a transcription factor, regulates the activities of the *DNA Methyltransferase 3A* (*DNMT3A*) and *DNMT3B* promoters by binding to their promoters in human cells.^33^ Epimutations in *SP3* lead to transcriptional repression and down‐regulate *DNMT3A* expression. The loss of *Dnmt3a* predisposes mouse hematopoietic stem cells (HSCs) to malignant transformation.^34^ *Dnmt3a*‐ablation HSCs give rise to myeloid malignancies and lymphoma.^34^ Therefore, further studies are needed to establish whether epimutations in *SP3* transform human lymphocytes to lymphoma.

In the present study, we created a CRISPR‐Cas9 platform that edits epigenetic marks and represses the expression of a targeted gene. We applied this platform to biomedical research, and revealed that normal human cells were transformed to cancer cells in vitro through epimutations.

## 2 RESULTS

### 2.1 System design

**FIGURE 1:**
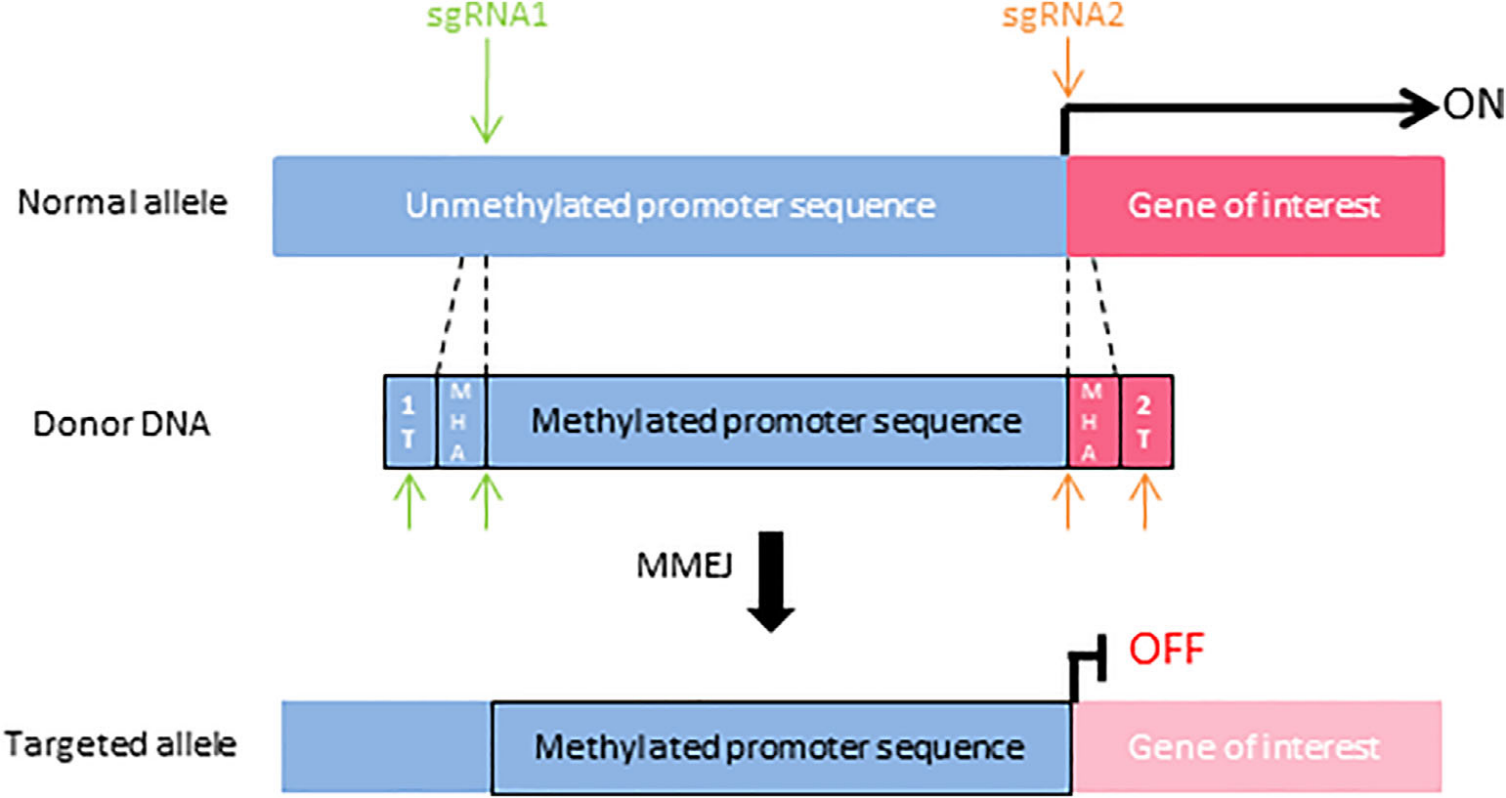
Design and characterization of the targeted DNA methylation system. The unmethylated promoter sequence between sgRNA1 and sgRNA2 is replaced with a methylated sequence through microhomology‐mediated end joining (MMEJ), thereby completely repressing transcription. 1 T, sgRNA1 target site; 2 T, sgRNA2 target site; green arrowheads, sgRNA1 cutting sites; orange arrowheads, sgRNA2 cutting sites

We designed a CRISPR/Cas9‐based method for targeted DNA methylation (Figure 1). The unmethylated promoter in the targeted gene was cut out by two sgRNA‐Cas9 complexes and replaced with the microhomology arm (MHA)‐harboring DNA fragment, which contained the methylated promoter and two sgRNA‐target sites (1 T and 2 T) of both ends, using MMEJ‐dependent integration, thereby repressing the transcription of the targeted gene. Since a previous study demonstrated that the replacement of a methylated 700‐bp sequence upstream of the transcription start site (TSS) with an unmethylated sequence induces powerful transcriptional activation,^22^ we considered the 700‐bp sequence upstream of the TSS to be crucial and, thus, targeted this region. The targeted region was amplified with PCR and then subjected to the insertion of DNA methylation with CpG methyltransferase (Donor DNA).

### 2.2 Construction of the targeted DNA methylation system

**FIGURE 2:**
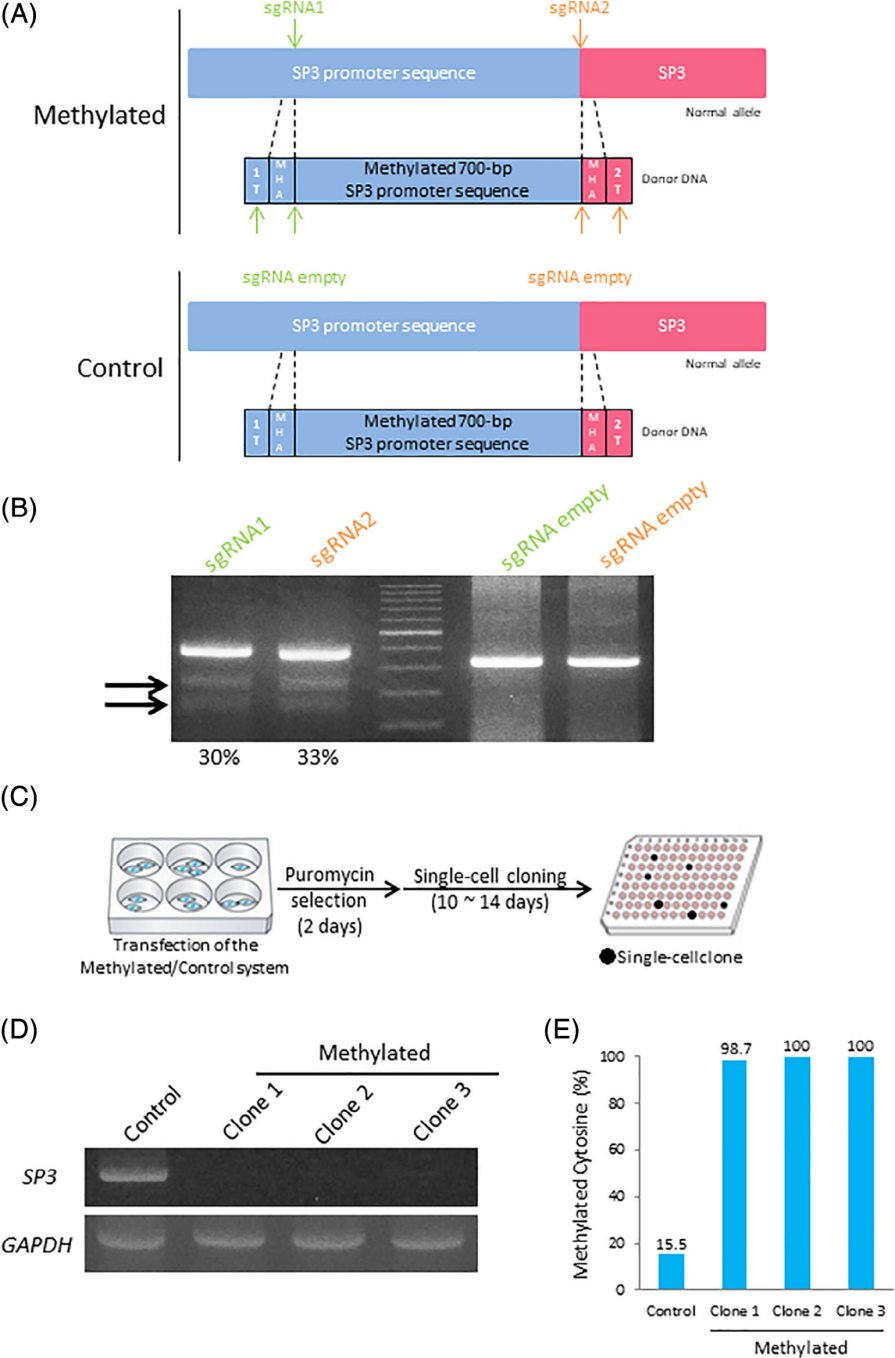
Targeted DNA methylation at the *SP3* promoter region. (A) Scheme of the targeted methylation system (*Methylated* and *Control*). (B) T7E1 assay for each sgRNA. Arrows: Size of cleaved fragments (also indicated below). %: Quantified editing efficiency. (C) Scheme of single‐cell cloning in HEK293 cells. (D) RT‐PCR analysis of *SP3* transcription. (E) Bisulfite sequencing analysis of the targeted *SP3* promoter region

We created two systems: the *Methylated* system to replace the unmethylated *SP3* promoter with the methylated 700‐bp *SP3* promoter and the *Control* system with no replacements (Figure 2A). The *Control* system consisted of sgRNA empty, Cas9, and a methylated donor DNA; therefore, methylated DNA was not integrated into the target genomic region.

To clarify whether the designed sgRNAs edit the targeted locus, we constructed pX459HypaCas9‐SP3 sgRNA1 and SP3 sgRNA2 vectors and transfected them into HEK293 cells. A T7 endonuclease I (T7E1) assay revealed that SP3 sgRNA1 and SP3 sgRNA2 cut the targeted loci at rates of 30 and 33%, respectively (Figure 2B). To establish whether the designed sgRNAs induced off‐target mutations, we selected the two highest potential off‐target sites of each sgRNA, which were ranked using CRISPOR (http://crispor.tefor.net/).^35^ We amplified the targeted sites by PCR and then subjected them to Sanger sequencing or a T7E1 assay. No mutations were detected in the potential off‐target sites (Figure S1).

To investigate whether our systems induced targeted DNA methylation, we generated single‐cell clones, which were transfected with the *Methylated* or *Control* system and selected with puromycin (Figure 2C). We observed the loss of *SP3* expression in single‐cell clones transfected with the *Methylated* system by reverse transcriptase PCR (RT‐PCR, Figure 2D). A bisulfite sequencing analysis confirmed an approximately 100% DNA methylation ratio at the targeted region in clones transfected with the *Methylated* system (Figure 2E, Figure S2). Collectively, these results demonstrated that our system induced targeted DNA methylation at a ratio of approximately 100% and may be used to repress the transcription of an endogenous gene.

### 2.3 Epimutations induce B‐cell lymphoma and are used to evaluate the risk of cancer

**FIGURE 3:**
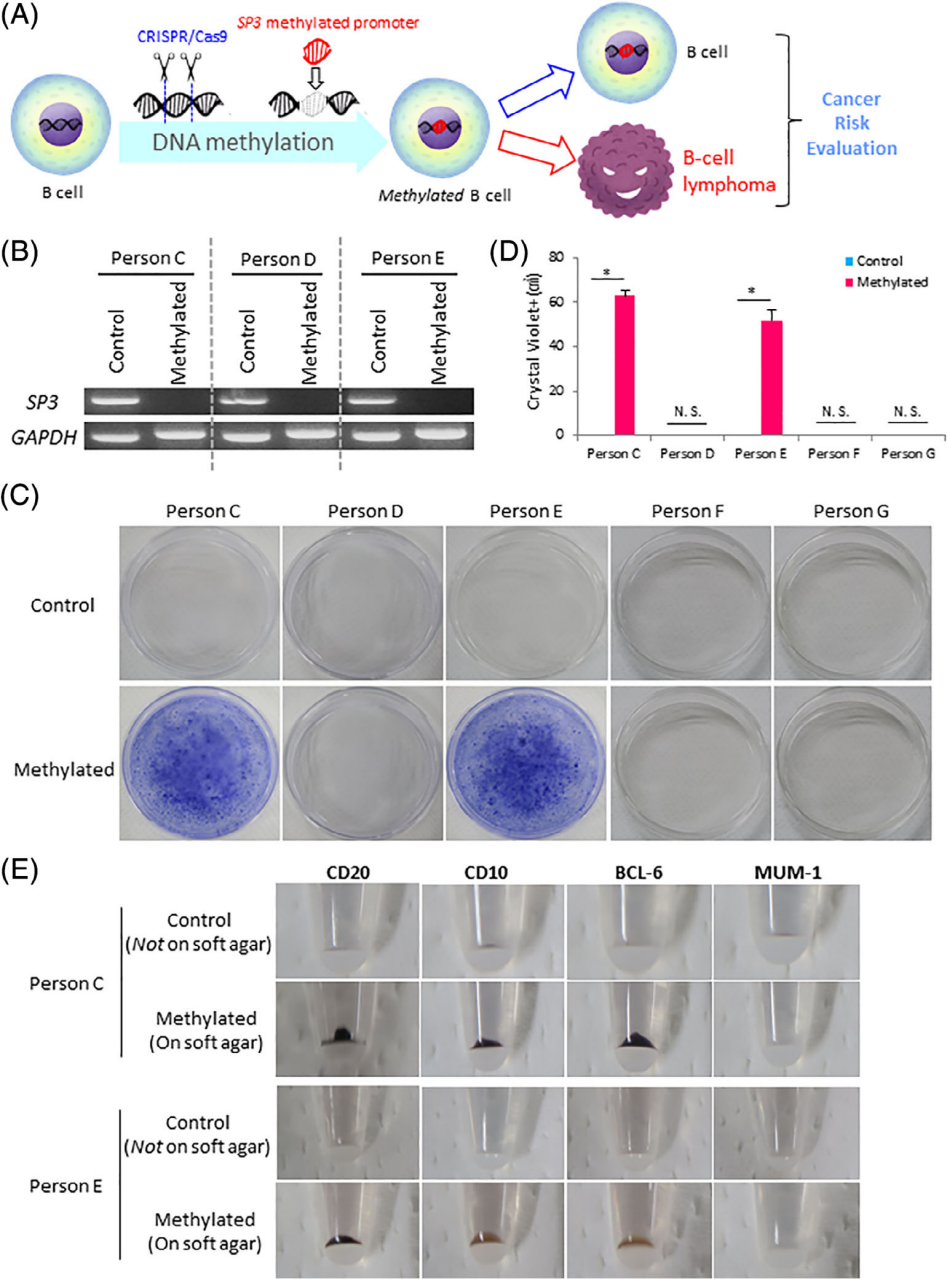
Epimutations induce B‐cell lymphoma and may be useful for evaluating the risk of cancer. (A) Scheme of DNA methylation and cancer risk evaluations. *Methylated* B. cells harboring the *SP3* epimutation were generated and subjected to the soft agar colony‐forming assay in order to assess whether *Methylated* B cells are B‐cell lymphoma. (B) RT‐PCR analysis of *SP3* transcription. (C) Soft agar colony‐forming assay. (D) Quantified area of crystal violet (+). Error bars indicate SD (*n* = 3), *P < .05 (two‐tailed *t*‐test). N. S., not significant. (E) Immunostaining for CD20, CD10, BCL‐6, and MUM‐1 (DAB, black‐brown precipitate). (B‐E) Persons C and E, died of B‐cell lymphoma; Persons D, F, and G, died of causes other than B‐cell lymphoma. Control, transfected with the *Control* system; Methylated, transfected with the *Methylated* system

We then investigated whether epimutations drive the transformation of B lymphocytes to B‐cell lymphoma and if they may be used to evaluate the risk of cancer. B cells were initially transduced with the targeted DNA methylation system, and *Methylated* B cells with epimutations at the *SP3* promoter region were generated (Figure 3A). *Methylated* B cells were subjected to soft agar colony‐forming assays to evaluate transformation to B‐cell lymphoma and their potential for cancer risk evaluations (Figure 3A). We prepared five types of B lymphocytes, which were derived from person C (died of B‐cell lymphoma), person D (died of causes other than B‐cell lymphoma), person E (died of B‐cell lymphoma), person F (died of causes other than B‐cell lymphoma), and person G (died of causes other than B‐cell lymphoma). The loss of *SP3* expression was observed in the three types of B cells transfected with the targeted methylation system (Figure 3B). The soft agar colony‐forming assay revealed that *Methylated* B cells derived from persons C and E acquired cancer cell properties, such as cell survival and proliferation, on soft agar in vitro (Figure 3C). The areas of crystal violet‐positive cells were approximately 62 and 51 cm^2^, respectively, on *Methylated* person C and person E cells, whereas these cells was not detected among *Methylated* person D, person F, and person G cells (Figure 3D). Clinically, B‐cell lymphoma was diagnosed based on the results of immunostaining with antibodies to CD20, CD10, BCL‐6, and MUM‐1.^36^ The result of CD20 (+), CD10 (+), BCL‐6 (+), and MUM‐1 (−) indicated that cells were germinal center B cell‐like (GCB) in B‐cell lymphoma.^36^ On the other hand, the result of CD20 (+), CD10 (−), BCL‐6 (−), and MUM‐1 (+) showed that cells were non‐GCB in B‐cell lymphoma.^36^ *Methylated* person C and E cells were immunolabeled for CD20, CD10, BCL‐6, and MUM‐1. Immunostaining revealed that *Methylated* person C and E cells were positive for CD20, CD10, and BCL‐6 and negative for MUM‐1 (Figure 3E), indicating that both of these cells were GCB in B‐cell lymphoma. The results shown in Figure 3C‐E correspond to the causes of death in persons C, D, E, F, and G. Collectively, these results demonstrated that epimutations at the *SP3* promoter region induced B‐cell lymphoma and may be used to evaluate the risk of cancer.

### 2.4 Knock‐in efficiency of our system

We investigated the knock‐in efficiency of our system. We transfected our system into HEK293 cells and subsequently performed puromycin selection. Nineteen single‐cell clones were generated in each *Methylated* and *Control* system. Six out of the 19 clones transfected with the *Methylated* system had methylated *SP3* promoters (Figure S3, left panel), as identified by bisulfite sequencing. Therefore, the knock‐in frequency of our system was approximately 31% (Figure S3, right panel).

### 2.5 Our system is applicable to other endogenous genes

To evaluate whether our system may be used to introduce DNA methylation at other unmethylated promoters, we focused on CDKN2A, the promoter of which is hypermethylated in various cancers.^37^, ^38^, ^39^, ^40^, ^41^ We constructed two systems: the *Methylated* system to replace the unmethylated *CDKN2A* promoter with the methylated 700‐bp *CDKN2A* promoter, and the Control system with no replacements (Figure S4A).

To investigate whether the designed sgRNAs edited the targeted regions, we constructed pX459HypaCas9‐CDKN2A sgRNA3 and sgRNA4 vectors and transfected them into HEK293 cells. T7E1 assays revealed that CDKN2A sgRNA3 and sgRNA4 cut the genome at 22% and 46%, respectively (Figure S4B). To clarify whether CDKN2A sgRNAs induced off‐target mutations, we selected the two highest potential off‐target sites of each sgRNA, which were ranked using CRISPOR (http://crispor.tefor.net/).^35^ We amplified the target sites by PCR and then subjected them to Sanger sequencing or a T7E1 assay. No mutations were noted in the potential off‐target sites (Figure S1).

We generated single‐cell clones transfected with the *Methylated* or *Control* system (Figure S4C). We confirmed the loss of *CDKN2A* expression by RT‐PCR (Figure S4D) and an approximately 100% DNA methylation ratio at the targeted region by bisulfite sequencing (Figure S4E) in single‐cell clones transfected with the *Methylation* system. Collectively, these results support the applicability of our system to other endogenous genes.

## 3 DISCUSSION

In the present study, we successfully developed a targeted DNA methylation system that enables an approximately 100% DNA methylation ratio at the targeted region. In this system, an unmethylated promoter is replaced with a methylated promoter through MMEJ.

As demonstrated herein, the replacement of an unmethylated promoter with a methylated promoter was sufficient to repress the expression of targeted genes. However, this result raised the question of how long the methylated status is maintained in cells. To answer this question, we cultured cells for 3 and 6 weeks after single‐cell cloning (Figure S5A). RT‐PCR showed the loss of *SP3* expression in cells cultured for 3 and 6 weeks (Figure S5B). Bisulfite sequencing revealed that the methylation status was maintained for 6 weeks (Figure S5C). Therefore, the methylation status is maintained for at least 6 weeks after single‐cell cloning.

DNMT1, which is essential for the maintenance of DNA methylation patterns during cellular division, is not influenced by the silencing of *SP3*. Therefore, edited DNA methylation is maintained during long‐term cultures.

The MMEJ strategy has been broadly used for biomedical research applications, such as the generation of knock‐in mice harboring a fluorescence protein,^24^, ^25^ gene therapy in mouse models,^26^ and disease modeling in human‐induced pluripotent stem cells,^27^ indicating that MMEJ is a precise and efficient knock‐in method. The present results also showed that the MMEJ‐based DNA methylation system may correctly and efficiently edit the epigenetic status of the targeted gene and induce epigenetic disease. Therefore, MMEJ‐based epigenome editing may be applicable to the induction and elucidation of epigenetic diseases.

Random donor DNA integrations at naturally occurring double‐stranded DNA (dsDNA) breaks have been observed when linear dsDNA is transfected into cells, albeit at low rates (~1%).^42^ This rare event may be completely reduced by using single‐stranded DNA (ssDNA) templates.^43^ Therefore, the usage of ssDNA templates may reduce random donor DNA integrations with the MMEJ strategy.

A similar study previously demonstrated that the combination of CRISPR dual cut and NHEJ‐based ligation enabled the replacement of an unmethylated promoter with a methylated promoter.^44^ Although this method showed low efficiency for introducing methylation (~1%)^44^ into a haploid cell line, our system introduced methylation at an efficiency of approximately 31% in a diploid cell line. The efficiency of introducing methylation into both alleles may be lower than that into a single allele. If this approach is used for diploid cell lines, efficiency (~1%) may markedly decrease. Therefore, our system is more suitable for introducing methylation into both alleles than this method.

Since MMEJ is an error‐phone mechanism, we need to confirm whether there are any alterations in the junction sequences of *Methylated* clones. All *Methylated* clones were analyzed by Sanger sequencing, which revealed no alterations in the junction sequences of *Methylated* clones (Figure S6).

The restoration of *SP3* expression has been proposed to suppress the cancer phenotype of lymphoma cells. We constructed a *SP3* expression vector (pEBMulti‐SP3) and transfected it into *Methylated* person C and person E cells. These cells were cultured in soft agar for 14 days (not 21 days) and then stained with crystal violet. The restoration of *SP3* expression did not suppress the cancer phenotype of lymphoma cells from *Methylated* person C or person E cells in the soft agar assay (Figure S7), suggesting that the cancer state, once established, cannot be reversed.

Epimutations trigger cancer initiation by mechanisms that currently remain unknown. The down‐regulation of *SP3* decreases *DNMT3A* expression levels, thereby diminishing DNMT3A‐mediated de novo DNA methylation. Somatic mutations in *DNMT3A* and its reduced enzymatic activity have been observed in acute monocytic leukemia.^45^ Reduced de novo DNA methylation may be related to cancer initiation through complex mechanisms, and, thus, further studies are needed to elucidate the underlying mechanisms.

Individual single‐nucleotide polymorphisms (SNPs) are one of the causes of genomic instability due to environmental exposure.^46^, ^47^ Genomic instability is a characteristic of most cancer cells.^48^ Artificial epimutation, instead of environmental exposure, is considered to be useful for assessing individual cancer risk.

The present results indicate that our targeted DNA methylation system enables the induction of B‐cell lymphoma, and will be useful for evaluating the risk of cancer.

## 4 MATERIALS AND METHODS

### 4.1 Vector construction

The CRISPR/Cas9 (*Streptococcus pyogenes* Cas9, SpCas9) plasmid was constructed using the pSpCas9 BB‐2A‐Puro (PX459) V2.0 system.^49^ SpCas9 was modified to HypaCas9^50^ with the Gibson assembly. Oligonucleotides for sgRNA templates were synthesized, annealed, and inserted into the corresponding vectors. pX459HypaCas9 vectors for the human SP3 and CDKN2A promoters, termed pX459HypaCas9‐SP3 sgRNA1, SP3 sgRNA2, CDKN2A sgRNA3, and CDKN2A sgRNA4, were constructed. All plasmids were verified using BigDye Terminator Kit version 3.1 (Applied Biosystems) and the ABI sequencer model 3130xl (Applied Biosystems). Oligonucleotide sequences are given in Table S1.

In order to construct donor DNAs, we amplified the 5′ genomic DNA of the human *SP3* and *CDKN2A* genes, 700‐bp upstream sequences that are registered as functional promoters in the Transcriptional Regulatory Element Database (TRED, http://rulai.cshl.edu/TRED),^51^, ^52^ from the genomic DNA of HEK293 cells with KOD ONE (Toyobo). After validation of the nucleotide sequences using BigDye Terminator Kit version 3.1 (Applied Biosystems) and ABI sequencer model 3130xl (Applied Biosystems), these genomic DNA fragments were subjected to the insertion of DNA methylation, which was performed with CpG Methyltransferase (M.SssI, NEB) according to the manufacturer's instructions. Methylated donor DNAs, termed the Methylated 700‐bp SP3 promoter sequence and Methylated 700‐bp CDKN2A promoter sequence, were constructed. Primer sequences are given in Table S2.

### 4.2 Cell culture, lipofection, and puromycin selection

HEK293 (JCRB9068) and B cells (purchased from JCRB, to avoid privacy exposure) were cultured at 37°C in 5% CO_2_ in Dulbecco's modified Eagle's medium (Nacalai Tesque) and RPMI640 (Nacalai Tesque), respectively, supplemented with 10% fetal bovine serum (HyClone), 100 U/mL of penicillin, and 100 μg/mL of streptomycin (Nacalai Tesque). PEIpro in vitro DNA transfection reagent (Polyplus) and Opti‐MEM (Life Technologies) were used for transfection in accordance with the manufacturers' instructions. The plasmid concentrations, cell numbers, and plates used were as follows: 150 ng for pX459HypaCas9‐empty, SP3 sgRNA1, SP3 sgRNA2, CDKN2A sgRNA3, and CDKN2A sgRNA4 vectors, and 150 ng for methylated SP3 and CDKN2A DNA templates into 1.0 × 10^5^ cells using a 24‐well plate. Puromycin selection was performed at a concentration of 1 μg/mL.

### 4.3 RNA isolation and reverse transcription

Total RNA was purified from HEK293 and B cells after transfection and puromycin selection with Qiazol reagent (Qiagen). One microgram of total RNA was used for the reverse transcription reaction with a ReverTra Ace PCR RT Kit (Toyobo), in accordance with the manufacturer's instructions. Primer sequences are given in Table S3.

### 4.4 T7E1 assay

Genomic DNA was extracted from 4‐day‐old HEK293 cells after transfection and puromycin selection. The target site was amplified using PCR with the appropriate primer set (Table S4). The PCR amplicon was purified using a DNA purification kit (Qiagen). Two hundred nanograms of each amplicon was diluted to 10 μL with 1× NEB2 buffer. The amplicon was denatured and rehybridized in a thermal cycler programmed for an incubation at 95°C for 10 minutes followed by 1 minute each at 85, 75, 65, 55, 45, 35, and 25°C. Four microliters of DDW, 0.5 μL 10 × NEB2 Buffer, and 0.5 μL 10 U/μL T7E1 (NEB) were added and the reactions were incubated at 37°C for 30 minutes. The resulting products were analyzed by electrophoresis on 2% agarose gels and visualized with Gel Red. The intensity of the bands of the PCR amplicon and cleavage products were measured using the ImageJ (NIH). Efficiency was calculated using the following formula: % gene modification = 100 × (1 – [1 – fraction cleaved]^1/2^).

### 4.5 Genomic PCR and off‐target analyses

Genomic DNA was extracted from HEK293 cells using the QIAamp DNA mini kit (Qiagen) in accordance with the manufacturer's instructions and subjected to PCR. Primer sequences are given in Tables S4 and S5.

We used CRISPOR (http://crispor.tefor.net/) to identify off‐target candidate sites for sgRNAs. The DNA sequencing of PCR‐amplified candidate sites was performed as described above. Primers were given in Table S5.

### 4.6 Single‐cell cloning

Four days after transfection and puromycin selection, HEK293 and B cells were dissociated and seeded into a single cell/well (96‐well plates) using a limited dilution; cells were diluted to 50 cells in 10 mL, and 100 μL was pipetted into each well of a 96‐well plate.^53^ 2 to 3 weeks after seeding, living clones were picked up and genomic DNA was extracted for PCR.

### 4.7 Immunocytochemistry

Staining with anti‐CD20, CD10, BCL‐6, and MUM‐1 antibodies was performed as described previously.^36^ Briefly, mouse anti‐CD20 (L26; dilution 1:200; Invitrogen), mouse anti‐CD10 (56C6; dilution 1:1; Invitrogen), mouse anti‐BCL‐6 (D‐8; dilution 1:75; Santa Cruz), and mouse anti‐MUM‐1 (MUM1p; dilution 1:10; Dako) antibodies were used as the primary antibody in a 1‐hour reaction at room temperature and a goat anti‐mouse IgG HRP conjugate (dilution 1:500; ProteinTech) was then used as the secondary antibody.

### 4.8 Soft agar colony‐forming assay

In the colony‐forming assay using soft agar culture, 4 days after transfection and puromycin selection, cells were suspended in RPMI1640 containing 0.3% agar (BioRad) and 10% FBS and layered on RPMI containing 0.5% agar and 10% FBS in a 10‐cm dish. The dishes were incubated at 37°C for 21 days in 5% CO_2_. Medium was added every 3 to 4 days. After 21 days, colonies were stained with 0.005% Crystal Violet (Sigma) and dried at room temperature.

### 4.9 Bisulfite sequencing

Genomic DNA was extracted from HEK293 cells as described above. Bisulfite conversion was performed with the EpiMark Bisulfite Conversion Kit (NEB) according to the manufacturer's instructions. Converted genomic DNA templates were subjected to genomic PCR using EpiMark Hot Start Taq DNA Polymerase (NEB). Amplified PCR products were verified using BigDye Terminator Kit version 3.1 (Applied Biosystems) and the ABI sequencer model 3130xl (Applied Biosystems). Primers were given in Table S4.

### 4.10 Statistical analysis

Data were analyzed using a two‐tailed Student's *t*‐test, with significant differences defined as *P* < .05.

## CONFLICT OF INTEREST

Shota Katayama is listed as an inventor in patent applications related to this work. The other authors declare no conflicts of interest.

## AUTHOR CONTRIBUTIONS

Shota Katayama designed and conceived the study. Shota Katayama performed all of the experiments and analyzed the data. Koichi Shiraishi, Naoki Gorai, and Masao Andou made suggestions for manuscript writing and experimental designs. Shota Katayama wrote the manuscript.

## ETHICS STATEMENT

This work was conducted with the approval of the Ethics Review Board of IMRA Japan Co., LTD. Koichi Shiraishi, Naoki Gorai, and Masao Andou are Ethics Review Board members.

## PEER REVIEW

The peer review history for this article is available at https://publons.com/publon/10.1002/ggn2.10040.

## Supporting information


**Appendix S1:** Supporting information.

**Figure S1** Off‐target analysis by Sanger sequencing, a, Off‐target analysis of SP3 (Left) and CDKN2A (Right) sgRNAs. Two potential off‐target candidate sites for each sgRNA were amplified from seven single‐cell clones by PCR. b, T7E1 assay of two potential off‐target candidate sites for each sgRNA. Genomic DNA was extracted from cells transfected with pX459HypaCas9‐sgRNA empty (−) or ‐each sgRNA (+). M: 100‐bp DNA ladder marker. Note that there were no bands of the expected size in the presence of off‐target mutations.
**Figure S2**: Bisulfite sequencing analysis of the targeted SP3 promoter region. Control (upper panel) and Methylated (lower panel) single‐cell clones were analyzed by bisulfite sequencing.
**Figure S3**: Knock‐in efficiency Numbers (left panel) and percentages (right panel) of knock‐in clones. Nineteen single‐cell clones were analyzed by bisulfite sequencing.
**Figure S4**: Targeted DNA methylation at the CDKN2A promoter region a, Schematic of the targeted methylation system (Methylated and Control). b, T7E1 assay for each sgRNA. Arrows: Size of cleaved fragments (also indicated below). %: Quantified editing efficiency. c, Scheme of single‐cell cloning in HEK293 cells. d, RT‐PCR analysis of CDKN2A transcription. e, Bisulfite sequencing analysis of the targeted CDKN2A promoter region.
**Figure S5**: DNA methylation over time a, Schematic representation of the long‐term experiment. b, RT‐PCR analysis of SP3 transcription. c, Bisulfite sequencing analysis of the targeted SP3 promoter region.
**Figure S6**: On‐target analysis by Sanger sequencing An on‐target analysis of SP3 loci. SP3 loci were amplified from 30 clones by PCR. Underlined areas indicate micro‐homologies.
**Figure S7**: The restoration of SP3 expression in the soft agar assay. a, Soft agar colony‐forming assay. b, Quantified area of crystal violet (+). Error bars indicate SD (n = 3). N. S., not significant.

**Table S1**: Oligonucleotide sequences.
**Table S2**: Primer sequences.
**Table S3**: Primer sequences.
**Table S4**: Primer sequences.
**Table S5**: Primer sequences.
