# Supplementary material for: A CRISPR/Cas9‐based method for targeted DNA methylation enables cancer initiation in B lymphocytes
Source: Adv Genet (Hoboken). 2021 Mar 11;2(1):e10040. doi: 10.1002/ggn2.10040 (PMC9744502; doi:10.1002/ggn2.10040)
Supplement: Supplementary file 2 — Figure S1 Off‐target analysis by Sanger sequencing, a, Off‐target analysis of SP3 (Left) and CDKN2A (Right) sgRNAs. Two potential off‐target candidate sites for each sgRNA were amplified from seven single‐cell clones by PCR. b, T7E1 assay of two potential off‐target candidate sites for each sgRNA. Genomic DNA was extracted from cells transfected with pX459HypaCas9‐sgRNA empty (−) or ‐each sgRNA (+). M: 100‐bp DNA ladder marker. Note that there were no bands of the expected size in the presence of off‐target mutations. Figure S2: Bisulfite sequencing analysis of the targeted SP3 promoter region. Control (upper panel) and Methylated (lower panel) single‐cell clones were analyzed by bisulfite sequencing. Figure S3: Knock‐in efficiency Numbers (left panel) and percentages (right panel) of knock‐in clones. Nineteen single‐cell clones were analyzed by bisulfite sequencing. Figure S4: Targeted DNA methylation at the CDKN2A promoter region a, Schematic of the targeted methylation system (Methylated and Control). b, T7E1 assay for each sgRNA. Arrows: Size of cleaved fragments (also indicated below). %: Quantified editing efficiency. c, Scheme of single‐cell cloning in HEK293 cells. d, RT‐PCR analysis of CDKN2A transcription. e, Bisulfite sequencing analysis of the targeted CDKN2A promoter region. Figure S5: DNA methylation over time a, Schematic representation of the long‐term experiment. b, RT‐PCR analysis of SP3 transcription. c, Bisulfite sequencing analysis of the targeted SP3 promoter region. Figure S6: On‐target analysis by Sanger sequencing An on‐target analysis of SP3 loci. SP3 loci were amplified from 30 clones by PCR. Underlined areas indicate micro‐homologies. Figure S7: The restoration of SP3 expression in the soft agar assay. a, Soft agar colony‐forming assay. b, Quantified area of crystal violet (+). Error bars indicate SD (n = 3). N. S., not significant. [file GGN2-2-e10040-s002.pdf]

Supplementary Figure 1

a

SP3 sgRNA1

Off-target site 1 CAGCAACAATAGCACAGAAG

7/7 CAGCAACAATAGCACAGAAG

Off-target site 2 CAACAGCATCAGCACAGAAA

7/7 CAACAGCATCAGCACAGAAA

SP3 sgRNA2

Off-target site 1 CACTCACAACCCACCCATTG

7/7 CACTCACAACCCACCCATTG

Off-target site 2 CTCTCTGAACCCGCCCATCG

7/7 CTCTCTGAACCCGCCCATCG

CDKN2A sgRNA3

Off-target site 1 TCCACGTTCCTGAAAATCAA

7/7 TCCACGTTCCTGAAAATCAA

Off-target site 2 CCCACTCCCATGAAAATCAA

7/7 CCCACTCCCATGAAAATCAA

CDKN2A sgRNA4

Off-target site 1 GCGGCCGGCGGCTGCAGAGA

7/7 GCGGCCGGCGGCTGCAGAGA

Off-target site 2 CCACCTGGCGGCTGCGGAGA

7/7 CCACCTGGCGGCTGCGGAGA

b

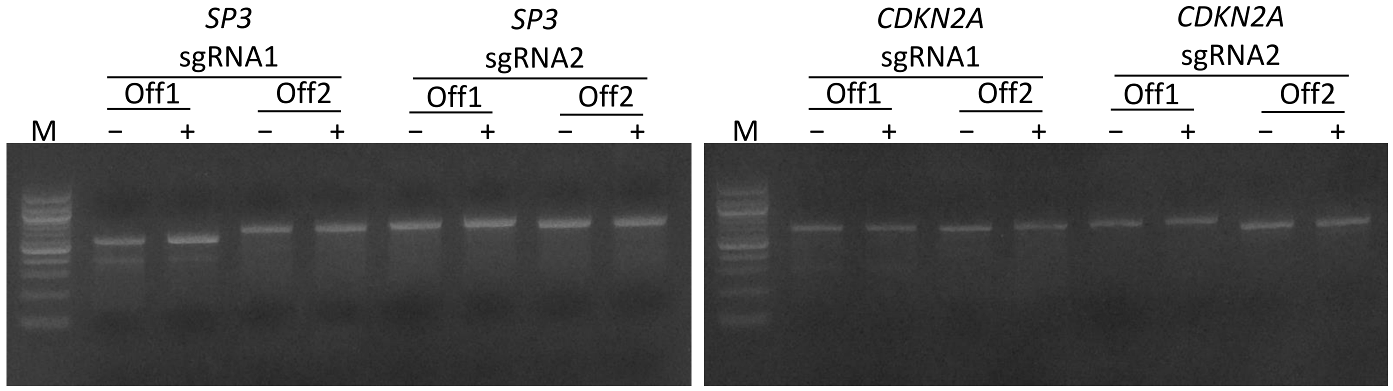

Supplementary Fig. 1 Off-target analysis by Sanger sequencing

a, Off-target analysis of *SP3* (Left) and *CDKN2A* (Right) sgRNAs. Two potential off-target candidate sites for each sgRNA were amplified from seven single-cell clones by PCR. **b**, T7E1 assay of two potential off-target candidate sites for each sgRNA. Genomic DNA was extracted from cells transfected with pX459HypaCas9-sgRNA empty (-) or -each sgRNA (+). M: 100-bp DNA ladder marker. Note that there were no bands of the expected size in the presence of off-target mutations.

Supplementary Figure 2

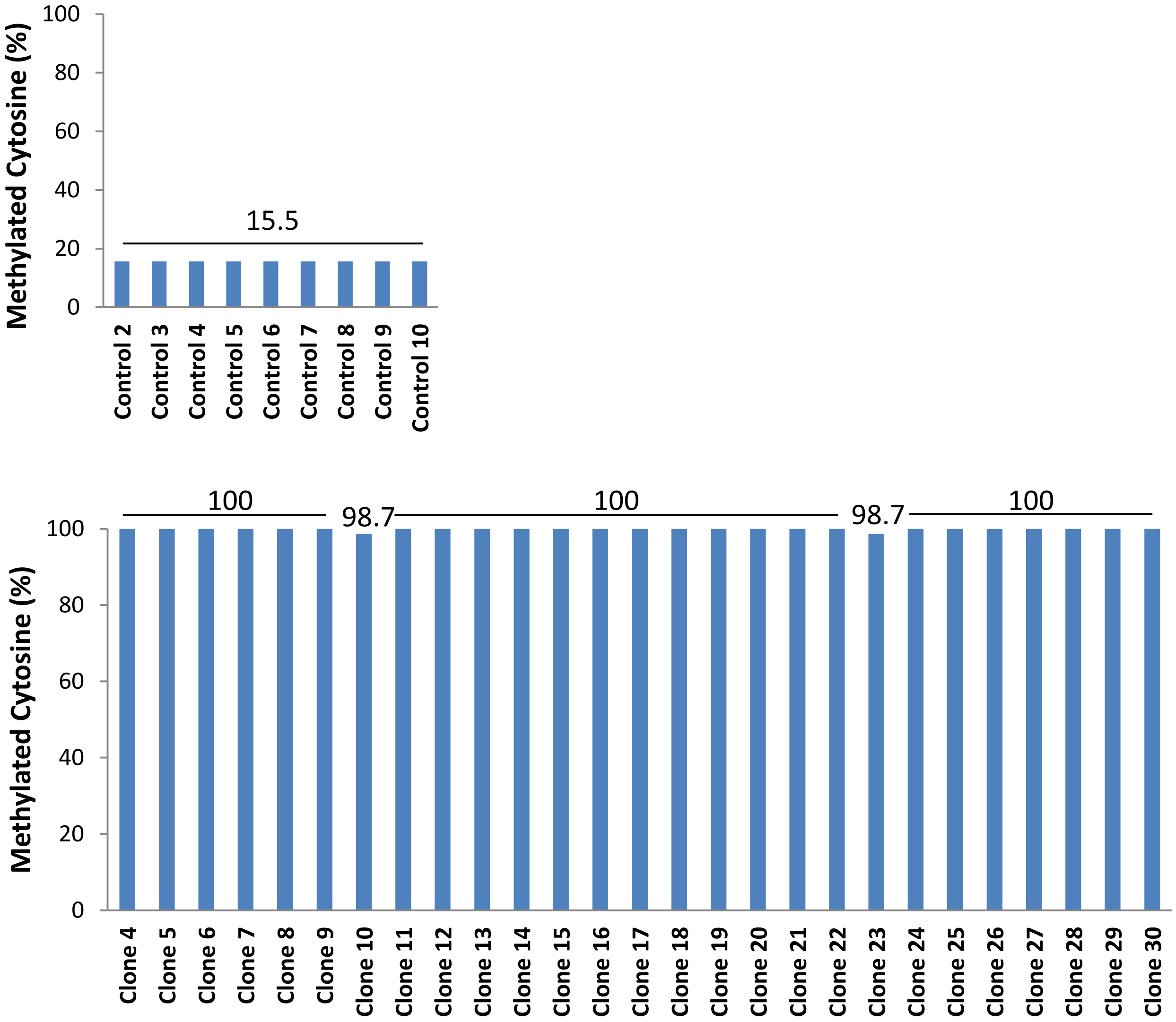

Supplementary Fig. 2 Bisulfite sequencing analysis of the targeted *SP3* promoter region. *Control* (upper panel) and *Methylated* (lower panel) single-cell clones were analyzed by bisulfite sequencing.

Supplementary Figure 3

	Numbers of knocked-in clones
Control	0 / 19
Methylated	6 / 19

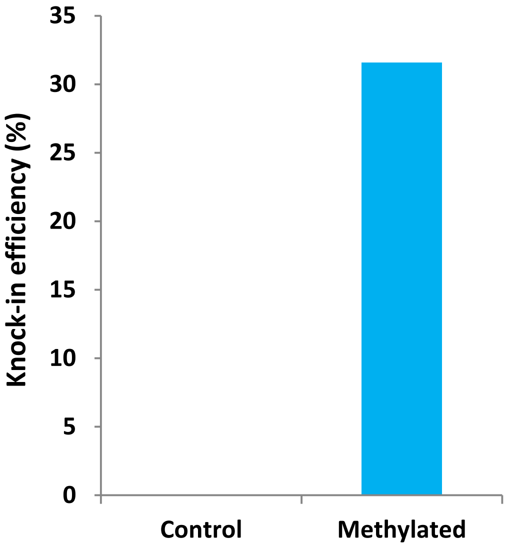

Supplementary Fig. 3 Knock-in efficiency
Numbers (left panel) and percentages (right panel) of knock-in clones. Nineteen single-cell clones were analyzed by bisulfite sequencing.

Supplementary Figure 4

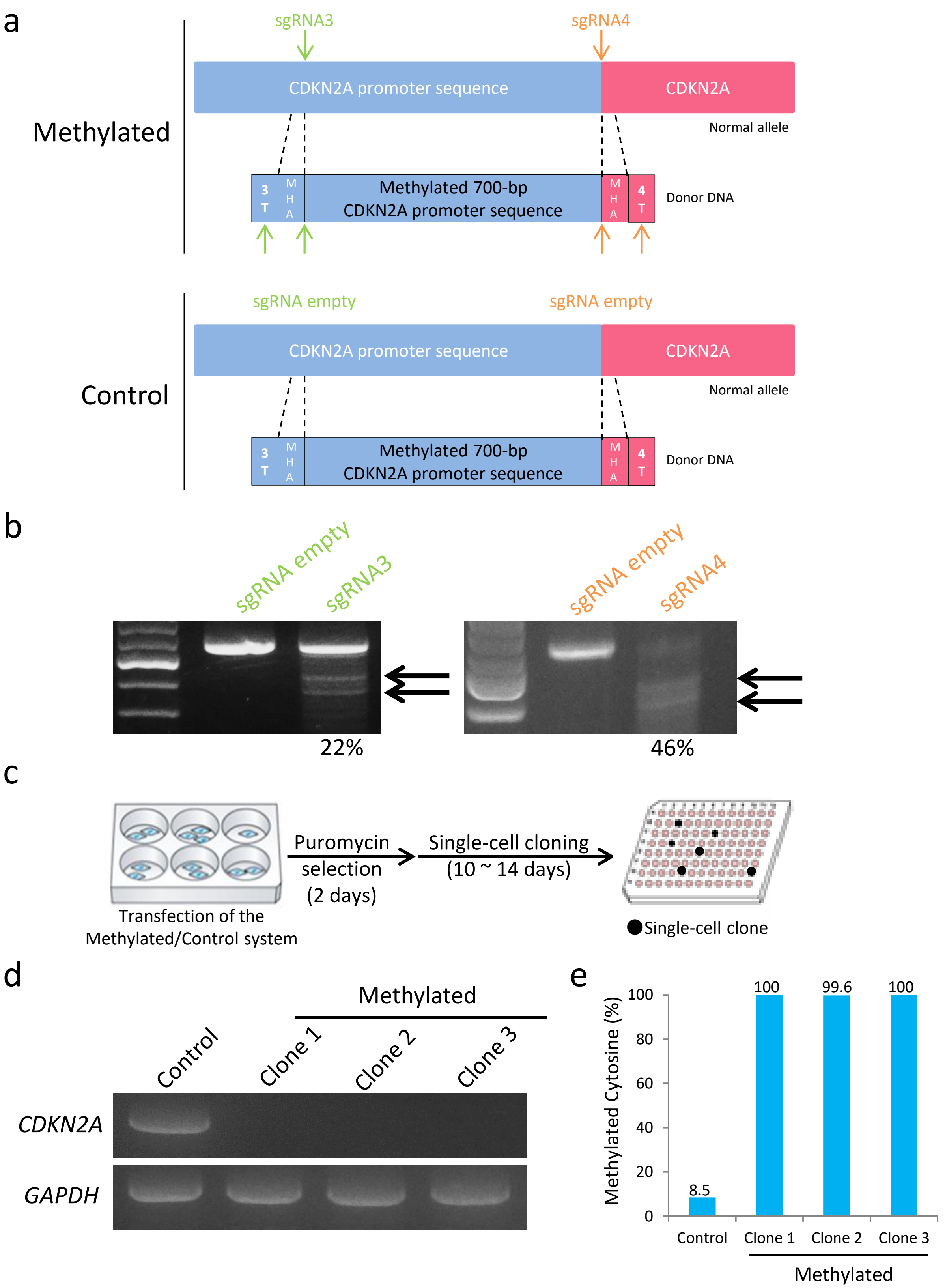

Supplementary Fig. 4 Targeted DNA methylation at the *CDKN2A* promoter region
a, Schematic of the targeted methylation system (*Methylated* and *Control*). **b**, T7E1 assay for each sgRNA. Arrows: Size of cleaved fragments (also indicated below). %: Quantified editing efficiency. **c**, Scheme of single-cell cloning in HEK293 cells. **d**, RT-PCR analysis of *CDKN2A* transcription. **e**, Bisulfite sequencing analysis of the targeted *CDKN2A* promoter region.

Supplementary Figure 5

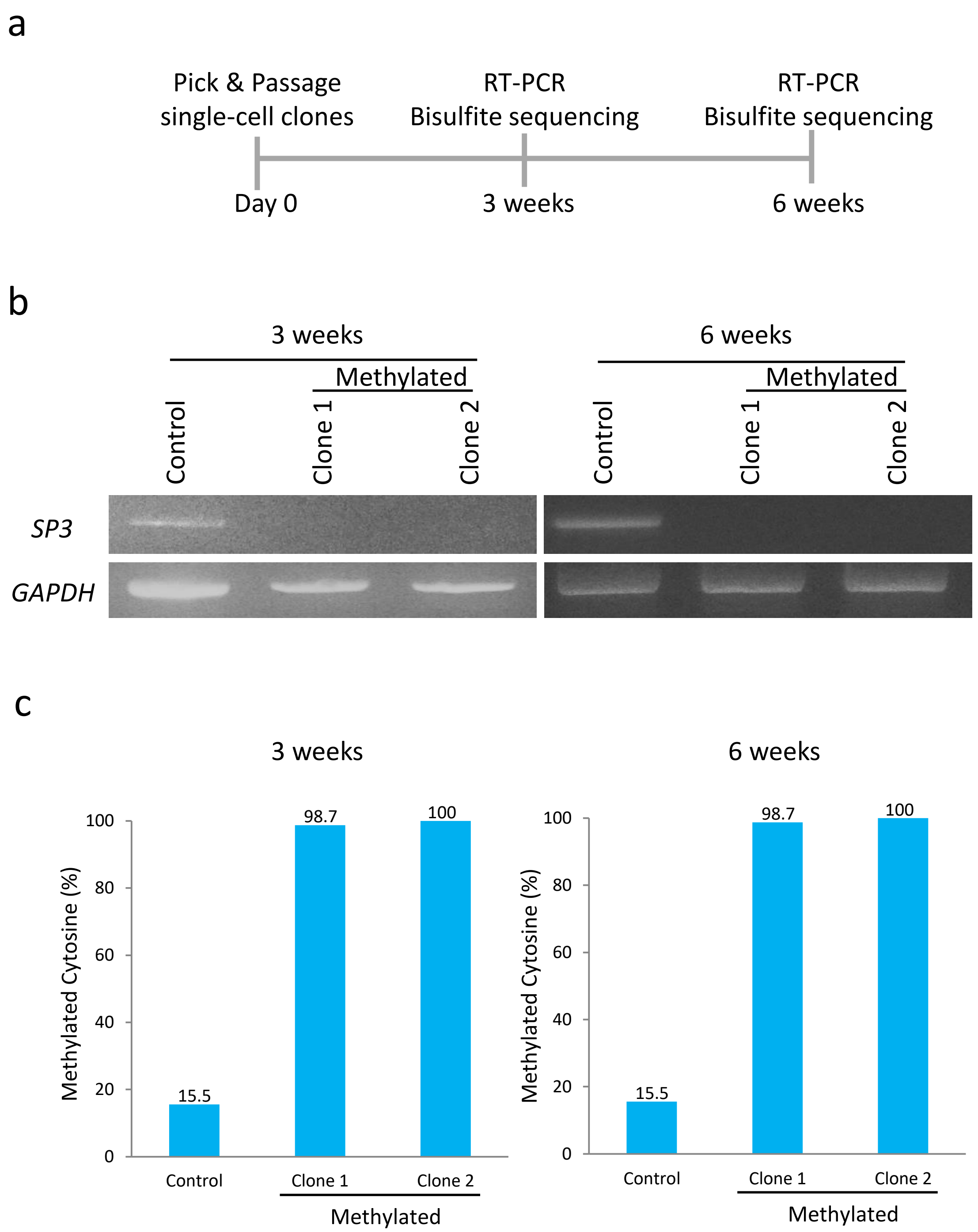

Supplementary Fig. 5 DNA methylation over time
a, Schematic representation of the long-term experiment. **b**, RT-PCR analysis of *SP3* transcription. **c**, Bisulfite sequencing analysis of the targeted *SP3* promoter region.

Supplementary Figure 6

SP3 5' junction

CGAGTATCTGCAGCAGCAGCAACAGCACGGAAACGGTGCG

SP3 3' junction

TCCCCCTGTCTCCCTCTCTGAACCCGCCCATTGGGGGTAG

30/30	Allele 1 CGAGTATCTGCAGCAGCAGCAACAGCACGGAAACGGTGCG<br>Allele 2 CGAGTATCTGCAGCAGCAGCAACAGCACGGAAACGGTGCG	Allele 1 TCCCCCTGTCTCCCTCTCTGAACCCGCCCATTGGGGGTAG<br>Allele 2 TCCCCCTGTCTCCCTCTCTGAACCCGCCCATTGGGGGTAG

Supplementary Fig. 6 On-target analysis by Sanger sequencing
An on-target analysis of *SP3* loci. *SP3* loci were amplified from 30 clones by PCR. Underlined areas indicate micro-homologies.

Supplementary Figure 7

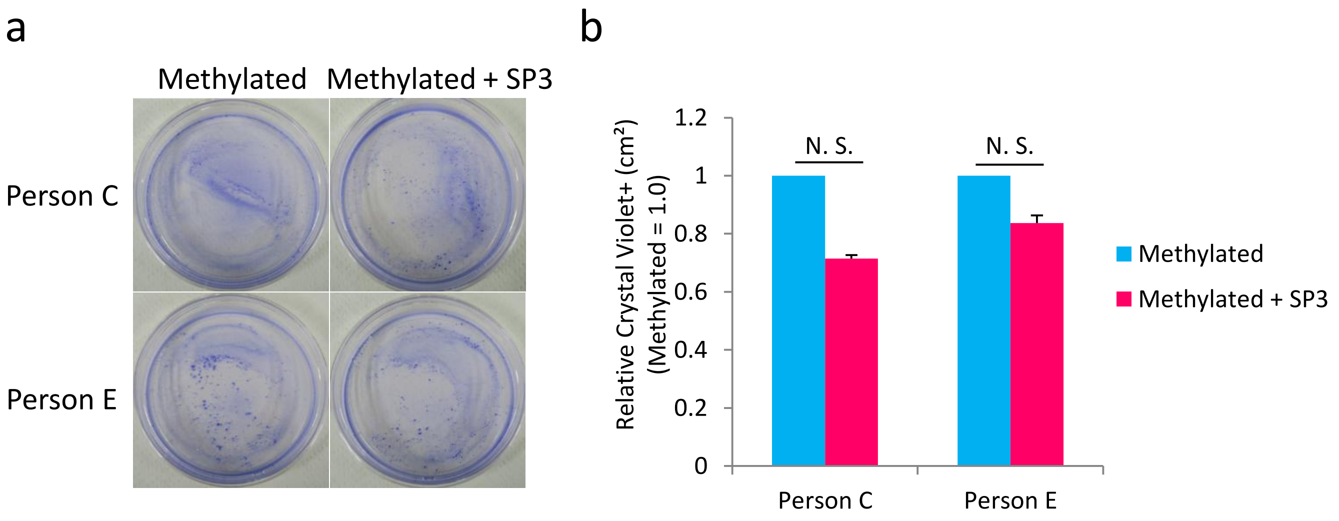

Supplementary Fig. 7 The restoration of *SP3* expression in the soft agar assay.
a, Soft agar colony-forming assay. **b**, Quantified area of crystal violet (+). Error bars indicate SD (n=3). N. S., not significant.
