# Supplementary material for: A CRISPR/Cas9‐based method for targeted DNA methylation enables cancer initiation in B lymphocytes
Source: Adv Genet (Hoboken). 2021 Mar 11;2(1):e10040. doi: 10.1002/ggn2.10040 (PMC9744502; doi:10.1002/ggn2.10040)
Supplement: Supplementary file 1 — Appendix S1: Supporting information. [file GGN2-2-e10040-s001.pdf]

ADVANCED GENETICS
Open Access

A CRISPR/Cas9-based method for targeted DNA methylation enables cancer initiation in B lymphocytes

Shota Katayama*, Koichi Shiraishi, Naoki Gorai, and Masao Andou

*Corresponding

Review timeline:

Date Submitted: 24-Aug-2020
Editorial Decision: 16-Oct-2020 Major Revision
Revision Received: 11-Dec-2020
Editorial Decision: 15-Jan-2021 Minor Revision
Revision Received: 01-Feb-2021
Accepted: 09-Feb-2021

Editor: Alison Liu

Initial Editorial Evaluation	25-Aug-2020

Summary

These authors indicate that they have developed a new system using two sgRNA-Cas9 complexes to cut off the promoter region of a cancer-related gene SP3 in cells and then replaces it with an exogenous DNA fragment of this promoter region with100% cytosine methylation. The DNA fragment has the microhomology arm (MHA), which allows MMEJ-dependent integration. The authors show that 100% methylation can effectively suppress the expression of SP3 and cause the lymphocytes to develop into B-cell lymphoma.

Scope

Do the research, methods or topics fit within the aims of this, or another journal?

This method is important because it allows potentially the introduction of any engineered DNA fragments with desired modifications (genetic mutations and epimutations) in the targeting sites of the genome precisely, while many other methods introduce the methylation modification by fusing Cas proteins with methylation enzymes and are hard to achieve the precision like this method. In addition, the correlation of 100% methylation using this method with the cancer development is significant functionally, which can prompt similar studies for cancer research and therapeutics.

1st Peer Review	20-Sep-2020 to 16-Oct-2020

Reviewer 1: Comments to the Author

This is an innovative approach to install epimutations through the use of genetic sequence editing. I would like the authors address these questions either through new experiments or discussion.

1.1) How stable is the methylation within the clone over cell divisions or cell passages, that is after the single-cell that has the actual MMEJ incorporation of the methylated DNA, how many times can the methylation replicated during DNA replication? It would be interesting to culture cells over passages and look at methylation over time.

1.2) Have the authors looked at off-target (Random) integration of the methylated DNA donor in the genome? If the authors have not, and it is a potential caveats, please state that in the discussion.

1.3) The authors stated that there are 6/19 clones with methylated promoter knockin (in Fig S2), but on in Fig 2e, only percentages of mC shown in Fig 2e. Were 3 out of the 6 selectively shown? Authors should provide percentages of all 6 clones.

Minor questions/suggestions:

1m1) The color in the schematic (such as the labeling “sgRNA2” is very light, so it is hard to read them. Please use a darker color

1m2) the Puromycin selection, where is the puromycin resistance marker located? On the donor DNA, on the Cas9 construct or the gRNA constructs?

1m3) Although described in the methods section, it is not obvious reading the maintext, how the methylated donor was generated. It might be beneficial to have one or two sentences in maintext for this.

Reviewer2: Comments to the Author

This paper presents a methodology to induce targeted methylation of genomic regions. It is novel and potentially interesting, but some details of the experiments need to be addressed.

2.1) In the first experiment when demonstrating that MMEJ-mediated DNA integration can be used to introduce methylation of target genomic region, the authors do not explain the control system. It's not clear if it involves a mock transfection with gRNA, Cas9 and a non-methylated donor DNA or just the un-transfected parental cells.

2.2) Since MMEJ is an error-prone mechanism the authors should address if there is any alteration of junction sequences in the methylated positive clones that could possibly contribute to the observed down-regulation of Sp3 expression.

2.3) The parental HEK293 cells which the authors use to establish this method is likely to be heterogeneous. It would be good to know if the authors have sampled single-cell clones of HEK293 to examine if different methylation levels on target genomic regions may pre-exist in the parental cells. More clones from the control and the experiment groups should be analyzed to score statistical significance to demonstrate this MMEJ-mediated-DNA integration method can actually impact a targeted-methylation level.

2.4) In figure 2e, the authors use the bisulfite sequence analysis to show the targeted methylation is almost 100%. Since the genomic DNA analyzed in this assay is harvested from cells cultured for 3 weeks after single-cell cloning, how could one explain that the hereditary methylation property? Especially the DNMT3A and DNMT3B are supposed to be downregulated by the silencing of SP3. Maybe some discussion can be added.

2.5) The experiment to demonstrate that epimutations at the SP3 promoter region induced B-cell lymphoma is encouraging, but the sample-size of patient cases is too small to make a sure claim.

2.6) Could the authors explain why the lymphoma cells from the patients do not already have a positive signal in the soft agar essay without SP3 methylation induction?

2.7) It would be convincing if the authors could show that the restoration of SP3 expression (e.g. by transfection) could suppress the cancer phenotype of the lymphoma cells from patient C and E in the soft agar assay.

Reviewer 3: Comments to the Author

As introduced in paper, targeted DNA methylation is an important approach to obtain the further understanding of transcriptional modulation and epigenetic diseases. Authors developed a novel method to study the risk of epigenetic diseases by CRISPR/Cas9-based DNA methylation system. This is an interesting study, potentially providing the theoretical guidance for understanding of cancer initiation in clinical application. I have several questions on the manuscript.

3.1) What kind of delivery system is used in this study to transfected HEK293 cells? Please specify.

3.2) Authors constructed 2 vectors to transduce HEK293 cells, but only used puro as selective marker to purify the infected cell populations. So how do you quantify the integration efficacy for two vectors and also please specify the steps of limited dilution approach in Single-cell cloning.

3.3) In Fig.2b, authors mentioned cutting efficiency of designed sgRNAs are only 30% and 33%, respectively. So how do you calculate the integration efficiency for two vectors? Checking SP3 expression through RT-PCR only can tell the survived clones have edited by CRISPR-cas9 system, and it's too difficult to tell that loss of SP3 expression is due to approximately 100% of targeted DNA methylation induction, maybe also caused by single cutting due to frameshift. Besides, only 3 clones are checked is tricky and artificial, thus, in order to solid the data, could you please check more clones, at least 30 clones.

3.4) About sgRNA induced off-target, whole genome sequencing is a typical approach to demonstrate. Prediction of potential off-target also may acceptable, but which needs more evidence to demonstrate, 2 sites for each sgRNA and only 7 clones are evaluated, apparently are not enough to conclude as "No mutation were detected in the potential off-target sites". If authors still want to conclude as above, please provide more data.

3.5) Authors used targeted DNA methylation system to evaluate the rick of cancer, which is good and very straightforward. But I do not understand to conclusion and how about the cells from person D? From the results, the Methylated person D cells are not expressed SP3 but also not induced as B cell lymphoma. What is the reason and please specify?

1st Editorial Decision	16-Oct-2020

Editor's understanding of the reviews

Reviewer #1 recommends Minor Revision

Reviewer #2 recommends Major Revision

Reviewer #3 recommends Major Revision

Editor Comments to Author: These authors indicate that they have developed a new system using two sgRNA-Cas9 complexes to cut off the promoter region of a cancer-related gene SP3 in cells and then replaces it with an exogenous DNA fragment of this promoter region with100% cytosine methylation. The DNA fragment has the microhomology arm (MHA), which allows MMEJ-dependent integration. The authors show that 100% methylation can effectively suppress the expression of SP3 and cause the lymphocytes to develop into B-cell lymphoma. This method is important because it allows potentially the introduction of any engineered DNA fragments with desired modifications (genetic mutations and epimutations) in the targeting sites of the genome precisely, while many other methods introduce the methylation modification by fusing Cas proteins with methylation enzymes and are hard to achieve the precision like this method. In addition, the correlation of 100% methylation using this method with the cancer development is significant functionally, which can prompt similar studies for cancer research and therapeutics.

However, to warrant the publication in the Advanced Genetics, the authors need to address several issues to clarify the novelty of the method and establish a strong conclusion of the cancer-induction effect. The reviewers questioned about potential off-target problems, low number of patients and cells tested, and the details of the method, etc. These reviews are constructive and need to be all addressed with additional information, discussion and proper numbers of experiments. Reviewer 2 suggested to restore the expression of SP3 to see if it can reduce the incident of cancer caused by 100% methylation in the SP3 promoter – this can help establish the correlation of methylation effect to the cancer, so it is also worthwhile to consider doing.

In addition, similar methods have been published recently in two papers, Nishiguchi et al. Nature Communications. 2020 (DOI: 10.1038/s41467-019-14181-3), and Alexander et al. BMC Biol. 2019 (DOI: 10.1186/s12915-019-0711-z). It is not clear why the authors did not cite these two papers, since the first author in this study is also a co-author in the Nature Communications paper. The authors need to cite previously papers even these works were done competitively during a similar period of time. Please explain the novelty of this method by comparing with the methods published in these two papers.

Author's Response to 1st Review	11-Dec-2020

Editor Comments to Author: … the authors need to address several issues to clarify the novelty of the method and establish a strong conclusion of the cancer-induction effect. The reviewers questioned about potential off-target problems, low number of patients and cells tested, and the details of the method, etc. These reviews are constructive and need to be all addressed with additional information, discussion and proper numbers of experiments. Reviewer 2 suggested to restore the expression of SP3 to see if it can reduce the incident of cancer caused by 100% methylation in the SP3 promoter – this can help establish the correlation of methylation effect to the cancer, so it is also worthwhile to consider doing.

Thank you for these comments. As requested by the Reviewers, we performed additional experiments.

In addition, similar methods have been published recently in two papers, Nishiguchi et al. Nature Communications. 2020 (DOI: 10.1038/s41467-019-14181-3), and Alexander et al. BMC Biol. 2019 (DOI: 10.1186/s12915-019-0711-z). It is not clear why the authors did not cite these two papers, since the first author in this study is also a co-author in the Nature Communications paper. The authors need to cite previously papers even these works were done competitively during a similar period of time. Please explain the novelty of this method by comparing with the methods published in these two papers.

Nishiguchi et al. has already been cited in the Discussion section (Ref. 40), which is a gene therapy paper on the replacement of mutated sequences with normal sequences, not epigenome editing. We explained the novelty of our method by comparing it with that reported by Alexander et al. in the Discussion section on page 9-10. (Did not explain why this method has 31% efficiency, compared to the #42 that has 1%? Also, Ref 40 (Nishiguchi et al.) should be cited in the Introduction).

Responses to the Reviewer 1:

We thank the Reviewer for the constructive comments. We performed additional experiments and made changes to the manuscript and figures.

This is an innovative approach to install epimutations through the use of genetic sequence editing. I would like the authors address these questions either through new experiments or discussion.
(1.1) How stable is the methylation within the clone over cell divisions or cell passages, that is after the single-cell that has the actual MMEJ incorporation of the methylated DNA, how many times can the methylation replicated during DNA replication? It would be interesting to culture cells over passages and look at methylation over time.

Thank you for this comment. We added the following paragraph to the Discussion section on page 9: "As demonstrated herein, the replacement of an unmethylated promoter with a methylated promoter was sufficient to repress the expression of targeted genes. However, this result raised the question of how long the methylated status was maintained in cells. To answer this question, we cultured cells for 3 and 6 weeks after single-cell cloning (Supplementary Fig. 5a). RT-PCR showed the loss of SP3 expression in cells cultured for 3 and 6 weeks (Supplementary Fig. 5b). Bisulfite sequencing revealed that the methylation status was maintained for 6 weeks (Supplementary Fig. 5c). Therefore, the methylation status was maintained for at least 6 weeks after single-cell cloning."

(1.2) Have the authors looked at off-target (Random) integration of the methylated DNA donor in the genome? If the authors have not, and it is a potential caveats, please state that in the discussion.

Thank you for this comment. We added the following sentences to the Discussion section on page 9: "MMEJ-based knock-in occurs when the targeted sequences in the genome and donor vector are correctly recognized and cut by CRISPR/Cas9. sgRNAs used in the present study did not induce off-target mutations (it is not clear why the authors suggest that without including references). Therefore, the off-target integration of methylated DNA did not occur."

(1.3) The authors stated that there are 6/19 clones with methylated promoter knockin (in Fig S2), but on in Fig 2e, only percentages of mC shown in Fig 2e. Were 3 out of the 6 selectively shown? Authors should provide percentages of all 6 clones.

We agree with this comment. We added the mC percentages of all 6 clones to Fig. 2e (clone 1~3) and Supplementary Fig. 2 (clone 4~6). (why not include the data of all 6 clones in Fig 2e?)

Minor questions/suggestions:
(1m1) The color in the schematic (such as the labeling "sgRNA2" is very light, so it is hard to read them. Please use a darker color

We used a darker color as suggested.

(1m2) the Puromycin selection, where is the puromycin resistance marker located? On the donor DNA, on the Cas9 construct or the gRNA constructs?

As we described in the Methods section (Vector construction), the pX459HypaCas9 vector harbors gRNA, HypaCas9, and a puromycin resistance marker.

(1m3) Although described in the methods section, it is not obvious reading the maintext, how the methylated donor was generated. It might be beneficial to have one or two sentences in maintext for this.

We added the following sentence to the main text on page 5: "The targeted region was amplified with PCR and then subjected to the insertion of DNA methylation with CpG Methyltransferase (Donor DNA)." (How do you know the methylation reaction worked perfectly, so the promoter fragments are 100% methylated?)

Responses to the Reviewer 2:
We thank the Reviewer for the constructive comments. We performed additional experiments and made changes to the manuscript and figures.

This paper presents a methodology to induce targeted methylation of genomic regions. It is novel and potentially interesting, but some details of the experiments need to be addressed.
2.1) In the first experiment when demonstrating that MMEJ-mediated DNA integration can be used to introduce methylation of target genomic region, the authors do not explain the control system. It's not clear if it involves a mock transfection with gRNA, Cas9 and a non-methylated donor DNA or just the un-transfected parental cells.

We appreciate the Reviewer's comment. We added the following sentence to the Results on page 5: "The Control system consisted of sgRNA empty, Cas9, and a methylated donor DNA; therefore, methylated DNA was not integrated into the target genomic region."

2.2) Since MMEJ is an error-prone mechanism the authors should address if there is any alteration of junction sequences in the methylated positive clones that could possibly contribute to the observed down-regulation of Sp3 expression.

Thank you for this comment. We randomly picked up single-cell clones and then performed RT-PCR. Clones that did not express SP3 were defined as Methylated clones. All Methylated clones were analyzed by Sanger and bisulfite sequencing. The Sanger sequencing analysis revealed no alterations in the junction sequences of Methylated clones (data not shown).

2.3) The parental HEK293 cells which the authors use to establish this method is likely to be heterogeneous. It would be good to know if the authors have sampled single-cell clones of HEK293 to examine if different methylation levels on target genomic regions may pre-exist in the parental cells. More clones from the control and the experiment groups should be analyzed to score statistical significance to demonstrate this MMEJ-mediated-DNA integration method can actually impact a targeted-methylation level.

Thank you for this comment. We performed bisulfite sequencing on 10 Control and 30 Methylated single-cell clones, and confirmed an approximately 100% DNA methylation ratio at the targeted region in Methylated clones (Fig. 2e, Supplementary Fig. 2). We added this result to Supplementary Fig. 2. The authors did not answer the question, which refer to the target genomic regions, not inserts.

2.4) In figure 2e, the authors use the bisulfite sequence analysis to show the targeted methylation is almost 100%. Since the genomic DNA analyzed in this assay is harvested from cells cultured for 3 weeks after single-cell cloning, how could one explain that the hereditary methylation property? Especially the DNMT3A and DNMT3B are supposed to be downregulated by the silencing of SP3. Maybe some discussion can be added.

We appreciate this comment. We added the following sentence to the Discussion section on page 9: “DNMT1, which is essential for the maintenance of DNA methylation patterns during cellular division, is not influenced by the silencing of SP3. Therefore, edited DNA methylation is maintained during long-term cultures.” (The reviewer asked about DNMT3A and DNMT3B, so the authors should indicate that DNMT3A and DNMT3B are for de nova DNA methylation)

2.5) The experiment to demonstrate that epimutations at the SP3 promoter region induced B-cell lymphoma is encouraging, but the sample-size of patient cases is too small to make a sure claim.

We appreciate this comment. We additionally prepared two types of B lymphocytes, which were derived from Person F (died of causes other than B-cell lymphoma) and Person G (died of causes other than B-cell lymphoma). The soft agar assay revealed that Methylated B cells derived from Persons F and G did not acquire cancer-cell properties (Figs. 3c and 3d). We added this result to Fig. 3c and Fig. 3d.

2.6) Could the authors explain why the lymphoma cells from the patients do not already have a positive signal in the soft agar essay without SP3 methylation induction?

As described in the Methods section, we purchased normal B cells derived from patients, not lymphoma cells. Normal B cells do not contain lymphoma cells. Therefore, we cannot observe a positive signal in the soft agar assay without the induction of SP3 methylation.

2.7) It would be convincing if the authors could show that the restoration of SP3 expression (e.g. by transfection) could suppress the cancer phenotype of the lymphoma cells from patient C and E in the soft agar assay.

Thank you for this comment. We constructed a SP3 expression vector (pEBMulti-SP3) and transfected it into Methylated person C and person E cells. These cells were cultured in soft agar for 14 days (not 21 days) and then stained with crystal violet. The results obtained are shown below.

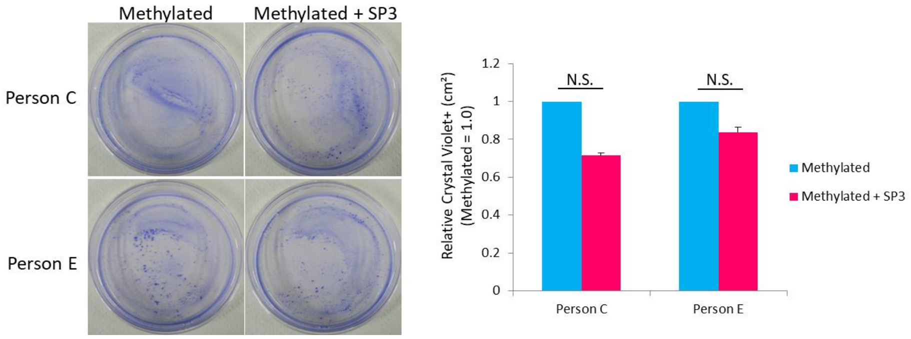

The restoration of SP3 expression did not suppress the cancer phenotype of lymphoma cells from Methylated person C and person E cells in the soft agar assay.
(The error bars are not properly presented. If the SP3 suppression caused cancer phenotype, with the expression of SP3, should the cancer be suppressed? If not, does that suggest that the correlation has not been established, or the cancer state cannot be reversed once induced? The authors should explain this result

Responses to the Reviewer 3:
We thank the Reviewer for the constructive comments. We performed additional experiments and changed the manuscript and figures.

As introduced in paper, targeted DNA methylation is an important approach to obtain the further understanding of transcriptional modulation and epigenetic diseases. Authors developed a novel method to study the risk of epigenetic diseases by CRISPR/Cas9-based DNA methylation system. This is an interesting study, potentially providing the theoretical guidance for understanding of cancer initiation in clinical application. I have several questions on the manuscript.
3.1) What kind of delivery system is used in this study to transfected HEK293 cells? Please specify.

Thank you for this comment. We used Lipofection and changed "Cell culture, DNA transfection, and Puromycin selection" to "Cell culture, Lipofection, and Puromycin selection" in the Methods section.

3.2) Authors constructed 2 vectors to transduce HEK293 cells, but only used puro as selective marker to purify the infected cell populations. So how do you quantify the integration efficacy for two vectors and also please specify the steps of limited dilution approach in Single-cell cloning.

Thank you for this comment. As requested by Reviewer 2, we added the following sentence to the Results section on page 5: "The Control system consisted of sgRNA empty, Cas9, and a methylated donor DNA; therefore, methylated DNA was not integrated into the target genomic region." As shown in Supplementary Fig. 3, we randomly picked up 19 single-cell clones of each system and performed bisulfite sequencing. The integration of methylated sequences was observed in 6 single-cell clones. Therefore, the integration efficacy of Methylated system was approximately 31%.
We added the following sentence to the Methods section (Single-cell cloning): "Four days after transfection and puromycin selection, HEK293 and B cells were dissociated and seeded into a single cell/well (96-well plates) using a limited dilution; cells were diluted to 50 cells in 10 ml, and 100 µl was pipetted into each well of a 96-well plate5."

3.3) In Fig.2b, authors mentioned cutting efficiency of designed sgRNAs are only 30% and 33%, respectively. So how do you calculate the integration efficiency for two vectors? Checking SP3 expression through RT-PCR only can tell the survived clones have edited by CRISPR-cas9 system, and it's too difficult to tell that loss of SP3 expression is due to approximately 100% of targeted DNA methylation induction, maybe also caused by single cutting due to frameshift. Besides, only 3 clones are checked is tricky and artificial, thus, in order to solid the data, could you please check more clones, at least 30 clones.

Thank you for this comment.
We performed a bisulfite sequencing analysis of 10 Control and 30 Methylated single-cell clones, and confirmed an approximately 100 % DNA methylation ratio at the targeted region in Methylated clones (Fig. 2e, Supplementary Fig. 2). We added this result to Supplementary Fig. 2.
We randomly picked up single-cell clones and then performed RT-PCR. Clones that did not express SP3 were defined as Methylated clones. All Methylated clones were analyzed by Sanger and bisulfite sequencing. The Sanger sequencing analysis revealed no alterations in the junction sequences of Methylated clones (data not shown).

3.4) About sgRNA induced off-target, whole genome sequencing is a typical approach to demonstrate. Prediction of potential off-target also may acceptable, but which needs more evidence to demonstrate, 2 sites for each sgRNA and only 7 clones are evaluated, apparently are not enough to conclude as "No mutation were detected in the potential off-target sites". If authors still want to conclude as above, please provide more data.

Thank you for this comment. Two sites for each sgRNA and 7 clones were sufficient to conclude that "No mutations were detected in the potential off-target sites" (Katayama et al., Life Science Alliance 3, 2020).
We additionally performed the T7E1 assay on cells transfected with pX459HypaCas9-sgRNA empty or -each sgRNA, and revealed that no mutations were detected in potential off-target sites (Supplementary Fig. 1b). We added the following to the Results section on page 5 and 7: "..then subjected them to Sanger sequencing or a T7E1 assay".

3.5) Authors used targeted DNA methylation system to evaluate the rick of cancer, which is good and very straightforward. But I do not understand to conclusion and how about the cells from person D? From the results, the Methylated person D cells are not expressed SP3 but also not induced as B cell lymphoma. What is the reason and please specify?

Thank you for this comment. We added the following paragraph to the Discussion section on page 10: "Individual single-nucleotide polymorphisms (SNPs) are one of the causes of genomic instability due to environmental exposure44,45. Genomic instability is a characteristic of most cancer cells46. Artificial epimutation, instead of environmental exposure, is considered to be useful for assessing individual cancer risk." We speculate that the reason why Methylated person D cells were not induced to B-cell lymphoma is individual SNPs.

We wish to express our thanks to the Reviewers for providing these highly constructive comments. With these additional modifications, we hope that the revised manuscript is now acceptable for publication. Thank you very much.

2nd Peer Review	14-Dec-2020 to 11-Jan-2021

Reviewer 1: Comments to the Author

The response to Reviewer 1 Q2 is not adequate in my opinion. It is because random donor DNA integrations irregardless of the specific CRISPR method have been observed when (linear double-stranded) DNA is transfected into cells (https://www.ncbi.nlm.nih.gov/pmc/articles/PMC6239417/, https://www.genscript.com/high-gene-knock-in-efficiency-and-reduced-off-target-integration-with-single-stranded-dna-hdr-template.html) . I think the authors should state the caveats if not wanting to conduct additional experiments. On top of that, off-target cutting by CRISPR can also occur, generating indels at off-target sites using this method of epigenome editing (as opposed to using dCas9-fused effector). Authors should also state this caveat if no whole-genome sequencing is performed to confirm no off-target indels.

The response to reviewer Q2 about sequencing junctional sequences should not state "data not shown". Authors should have a supplementary figure showing the sequencing trace since the experiment has already been performed.

Reviewer 2: Comments to the Author

I am satisfied with the authors' revision.

2nd Editorial Decision	15-Jan-2021

Editor's understanding of the reviews

Reviewer #1 recommends Minor Revision

Reviewer #2 recommends Accept

The authors have conducted some experiments required by reviewers, which has improved the paper significantly. The strength of this paper is in its application of an established method to cancer research. However, some minor issues still need to be clarified before we can accept the paper for publication.

E1) Reviewer #1 have additional questions that still need to be addressed.
E2) The references 39-42 about the MMEJ strategy need to be cited in Introduction.
E3) In the Discussion section, the author mentioned the integration efficiency was 31% compared to the published paper (~1%) but did not offer an explanation. The author claimed that the published paper (#42) worked only for one gene, however, this is not an accurate statement since it was indicated in that paper that the study on one gene was for the proof of concept, and it does not mean that it only worked on one gene. The authors in this paper only worked on one gene SP3.
E4) For this statement, "MMEJ-based knock-in occurs when the targeted sequences in the genome and donor vector are correctly recognized and cut by CRISPR/Cas9. sgRNAs used in the present study did not induce off-target mutations. Therefore, the off-target integration of methylated DNA did not occur.", please cite published papers related to this conclusion.
E5) Regarding this statement, "The targeted region was amplified with PCR and then subjected to the insertion of DNA methylation with CpG Methyltransferase (Donor DNA).", can you please explain how you confirm that the promoter fragments are 100% methylated?
E6) Regarding this statement, "DNMT1, which is essential for the maintenance of DNA methylation patterns during cellular division, is not influenced by the silencing of SP3. Therefore, edited DNA methylation is maintained during long-term cultures.", the reviewer asked about DNMT3A and DNMT3B, so the authors should indicate that DNMT3A and DNMT3B are for de nova DNA methylation, but not for maintenance.
E7) Regarding your response, "Thank you for this comment. We constructed a SP3 expression vector (pEBMulti-SP3) and

transfected it into Methylated person C and person E cells. These cells were cultured in soft agar for 14 days (not 21 days) and then stained with crystal violet. The results obtained are shown below. The restoration of SP3 expression did not suppress the cancer phenotype of lymphoma cells from Methylated person C and person E cells in the soft agar assay", the error bars are missing on blue bars, does that mean that they are zero, or extremely small? If so, please indicate that in the figure legend. It is interesting that with the expression of SP3, the cancer was not suppressed. Does that suggest that the cancer state, once established, cannot be reversed? The authors should explain this result in the Discussion.

Also, the Materials and Methods section should be included in the main text in your revised manuscript. Overall, the paper can be still improved in writings with a better explanation of the rationale in Introduction and the use of this method for cancer research in Discussion.

Author's Response to 2nd Review	01-Feb-2021

We appreciate the informative comments of the Reviewer. As requested, we modified the text and supplementary figures accordingly. Point-by-point responses to the Reviewer's comments are listed below. We wish to express our thanks to the Editor and Reviewer for their highly constructive comments. With these additional modifications, we hope that the revised manuscript will now be considered acceptable for publication.

Editor Comments to Author:

The authors have conducted some experiments required by reviewers, which has improved the paper significantly. The strength of this paper is in its application of an established method to cancer research. However, some minor issues still need to be clarified before we can accept the paper for publication.

(E1) Reviewer #1 have additional questions that still need to be addressed.

Answers to these questions are provided below.

(E2) The references 39-42 about the MMEJ strategy need to be cited in Introduction.

The manuscript was revised accordingly.

(E3) In the Discussion section, the author mentioned the integration efficiency was 31% compared to the published paper (~1%) but did not offer an explanation. The author claimed that the published paper (#42) worked only for one gene, however, this is not an accurate statement since it was indicated in that paper that the study on one gene was for the proof of concept, and it does not mean that it only worked on one gene. The authors in this paper only worked on one gene SP3.

Thank you for this comment. We revised the following paragraph in the Discussion section on page 10: "A similar study previously demonstrated that the combination of CRISPR dual cut and NHEJ-based ligation enabled the replacement of an unmethylated promoter with a methylated promoter44. Although this method showed low efficiency for introducing methylation (~1%)44 into a haploid cell line, our system introduced methylation at an efficiency of approximately 31% in a diploid cell line. The efficiency of introducing methylation into both alleles may be lower than that into a single allele. If this approach is used for diploid cell lines, efficiency (~1%) may markedly decrease. Therefore, our system is more suitable for introducing methylation into both alleles than this method."

(E4) For this statement, "MMEJ-based knock-in occurs when the targeted sequences in the genome and donor vector are correctly recognized and cut by CRISPR/Cas9. sgRNAs used in the present study did not induce off-target mutations. Therefore, the off-target integration of methylated DNA did not occur.", please cite published papers related to this conclusion.

Thank you for this comment. We replaced the former paragraph with the following one in the Discussion section on page 9: "Random donor DNA integrations at naturally occurring double-stranded DNA (dsDNA) breaks have been observed when linear dsDNA is transfected into cells, albeit at low rates (~1%)42. This rare event may be completely reduced by using single-stranded DNA (ssDNA) templates43. Therefore, the usage of ssDNA templates may reduce random donor DNA integrations with the MMEJ strategy."

(E5) Regarding this statement, "The targeted region was amplified with PCR and then subjected to the insertion of DNA methylation with CpG Methyltransferase (Donor DNA).", can you please explain how you confirm that the promoter fragments are 100% methylated?

We did not investigate whether PCR fragments were approximately 100% methylated after the treatment with CpG methyltransferase. However, we confirmed that the targeted promoter region was approximately 100% methylated in cells. Therefore, this does not necessarily need to be confirmed at that stage.

(E6) Regarding this statement, “DNMT1, which is essential for the maintenance of DNA methylation patterns during cellular division, is not influenced by the silencing of SP3. Therefore, edited DNA methylation is maintained during long-term cultures.”, the reviewer asked about DNMT3A and DNMT3B, so the authors should indicate that DNMT3A and DNMT3B are for de nova DNA methylation, but not for maintenance.

Reviewer 2 asked about the hereditary methylation property, which involves the maintenance of DNA methylation (DNMT1), not de novo DNA methylation (DNMT3A and DNMT3B). Reviewer 2 was satisfied with our revision.

(E7) Regarding your response, “Thank you for this comment. We constructed a SP3 expression vector (pEBMulti-SP3) and transfected it into Methylated person C and person E cells. These cells were cultured in soft agar for 14 days (not 21 days) and then stained with crystal violet. The results obtained are shown below. The restoration of SP3 expression did not suppress the cancer phenotype of lymphoma cells from Methylated person C and person E cells in the soft agar assay”, the error bars are missing on blue bars, does that mean that they are zero, or extremely small? If so, please indicate that in the figure legend. It is interesting that with the expression of SP3, the cancer was not suppressed. Does that suggest that the cancer state, once established, cannot be reversed? The authors should explain this result in the Discussion.

We added the following paragraph to the Discussion section on page 10: “The restoration of SP3 expression has been proposed to suppress the cancer phenotype of lymphoma cells. We constructed a SP3 expression vector (pEBMulti-SP3) and transfected it into Methylated person C and person E cells. These cells were cultured in soft agar for 14 days (not 21 days) and then stained with crystal violet. The restoration of SP3 expression did not suppress the cancer phenotype of lymphoma cells from Methylated person C or person E cells in the soft agar assay (Supplementary Fig. 7), suggesting that the cancer state, once established, cannot be reversed.”

As shown in Supplementary Fig. 7b, blue bars (Methylated) are 1.0. Reviewer 2 was satisfied with our revision.

Also, the Materials and Methods section should be included in the main text in your revised manuscript.

The manuscript was revised accordingly.

Reviewer 1 : Comments to the Author

The response to Reviewer 1 Q2 is not adequate in my opinion. It is because random donor DNA integrations irregardless of the specific CRISPR method have been observed when (linear double-stranded) DNA is transfected into cells (https://www.ncbi.nlm.nih.gov/pmc/articles/PMC6239417/, https://www.genscript.com/high-gene-knock-in-efficiency-and-reduced-off-target-integration-with-single-stranded-dna-hdr-template.html). I think the authors should state the caveats if not wanting to conduct additional experiments.

Thank you for this comment. We replaced the former paragraph with the following one in the Discussion section on page 9: “Random donor DNA integrations at naturally occurring double-stranded DNA (dsDNA) breaks have been observed when linear dsDNA is transfected into cells, albeit at low rates (~1%)42. This rare event may be completely reduced by using single-stranded DNA (ssDNA) templates43. Therefore, the usage of ssDNA templates may reduce random donor DNA integrations with the MMEJ strategy.”

On top of that, off-target cutting by CRISPR can also occur, generating indels at off-target sites using this method of epigenome editing (as opposed to using dCas9-fused effector). Authors should also state this caveat if no whole-genome sequencing is performed to confirm no off-target indels.

As shown in Supplementary figure 1, no mutations were detected in the potential off-target sites.

The response to reviewer Q2 about sequencing junctional sequences should not state "data not shown". Authors should have a supplementary figure showing the sequencing trace since the experiment has already been performed.

We added the following paragraph to the Discussion section on page 10: “Since MMEJ is an error-phone mechanism, we need to confirm whether there are any alterations in the junction sequences of Methylated clones. All Methylated clones were

analyzed by Sanger sequencing, which revealed no alterations in the junction sequences of Methylated clones (Supplementary Fig. 6)."

We added Supplementary Figure 6 to the Supplementary Figures file.

We wish to express our thanks to the Reviewers for providing these highly constructive comments. With these additional modifications, we hope that the revised manuscript is now acceptable for publication. Thank you very much.

Final Decision	09-Feb-2021

The authors did a great job revising the paper. The article is accepted subject to formatting recommendations.
